# Collective Interactions of Quantum-Confined Excitons in Halide Perovskite Nanocrystal Superlattices

**DOI:** 10.1021/acsnano.4c12509

**Published:** 2024-12-26

**Authors:** Shai Levy, Orr Be’er, Saar Shaek, Alexey Gorlach, Einav Scharf, Yonatan Ossia, Rotem Liran, Kobi Cohen, Rotem Strassberg, Ido Kaminer, Uri Banin, Yehonadav Bekenstein

**Affiliations:** † Department of Materials Science and Engineering, Technion – Israel Institute of Technology, Haifa 32000, Israel; ‡ The Solid-State Institute, Technion – Israel Institute of Technology, Haifa 32000, Israel; § Institute of Chemistry and the Center for Nanoscience and Nanotechnology, The Hebrew University of Jerusalem, Jerusalem 91904, Israel

**Keywords:** nanocrystals, lead halide perovskites, superlattices, nanocrystal coupling, superfluorescence, quantum confinement

## Abstract

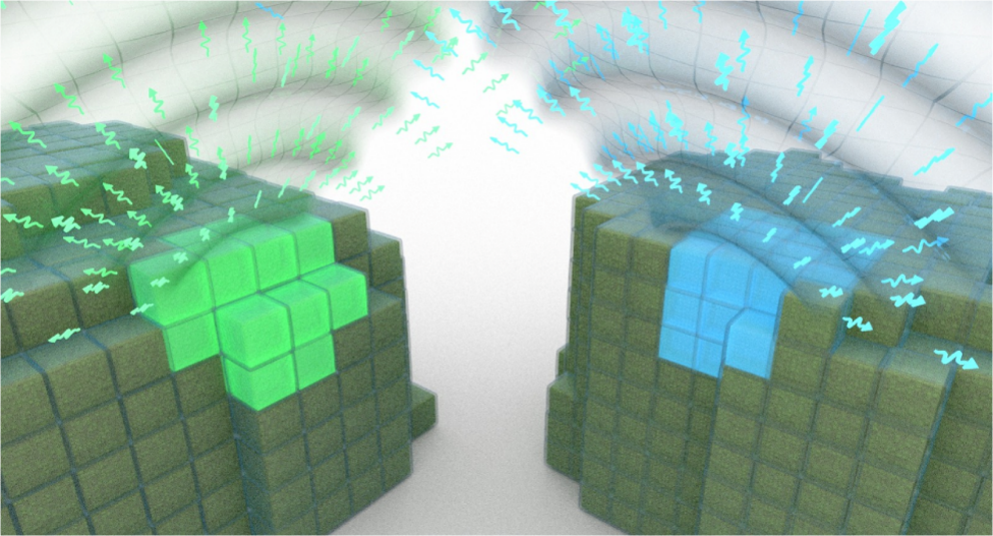

Collective optical properties can emerge from an ordered ensemble of emitters due to interactions between the individual units. Superlattices of halide perovskite nanocrystals exhibit collective light emission, influenced by dipole–dipole interactions between simultaneously excited nanocrystals. This coupling changes both the emission energy and rate compared to the emission of uncoupled nanocrystals. We demonstrate how quantum confinement governs the nature of the coupling between the nanocrystals in the ensemble. The extent of confinement is modified by controlling the nanocrystal size or by compositional control over the Bohr radius. In superlattices made of weakly confined nanocrystals, the collective emission is red-shifted with a faster emission rate, showing the key characteristics of superfluorescence. In contrast, the collective emission of stronger quantum-confined nanocrystals is blue-shifted with a slower emission rate. Both types of collective emission exhibit correlative multiphoton emission bursts, showing distinct photon bunching emission statistics. The quantum confinement changes the preferred alignment of transition dipoles within the nanocrystal and switches the relative dipole orientation between neighbors, resulting in opposite collective optical behaviors. Our results extend these collective effects to relatively high temperatures and provide a better understanding of exciton interactions and collective emission phenomena at the solid state.

The characteristics of a typical emitter are dictated by Fermi’s golden rule, with the transition rate proportional to the oscillator strength. However, in the case of multiple interacting emitters, this premise is modified in a nontrivial manner according to the nature of the collective interaction. In the case of several identical emitters located within a small volume, coherent collective coupling through common vacuum modes of the electromagnetic field may result in a faster emission rate by a process called superradiance, first described by Dicke in the 1950s.^1^ In this process, an ensemble of emitters behave as one large transition dipole with an oscillator strength proportional to the number of coupled emitters N.^2,3^ Dicke superradiance is commonly observed in dense atomic gases^4^ and recently reported in solid-state semiconductor systems.^5−7^ However, many semiconductor systems deviate from the ideal superradiance model framework due to dipole–dipole interactions between the emitters. Herein, we report the effects of quantum confinement on the dipole–dipole-influenced collective emission of perovskite nanocrystal (NC) ensembles. We control the type of dipole–dipole coupling by changing either the nanocrystal size or the halide composition, resulting in vastly different modes of correlative collective light emissions both spectrally and temporally.

Insights into the collective emission resulting from dipole–dipole interactions between emitters are found in the exciton interaction theory, developed by Kasha in the 1960s.^8,9^ When several emitters in proximity are simultaneously excited, Coulomb interactions between the transition dipoles of the neighboring emitters are possible. As a result of these dipole–dipole interactions, the system experiences an energy splitting by the value of the dipole–dipole interaction energy *J*_C_. In one of the coupled states, the transition dipoles are aligned in the same direction (triplet for a dimer), while the other state has the transition dipoles in opposite directions (singlet for a dimer). The state with aligned transition dipoles has an oscillator strength proportional to the number of interacting emitters, therefore showing characteristics similar to those of superradiance. For the case of a dimer, the dipole–dipole interaction energy is given by^9^
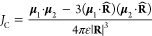
1where **μ**_i_ is the transition dipole, **R̂** is the vector connecting the two transition dipoles, and ε is the dielectric coefficient of the surrounding medium. In cases with a negative interaction (*J*_C_ < 0, “head-to-tail” interaction), the triplet state has lower energy than the excited state of an individual emitter. Ground-triplet exciton transitions are optically allowed, while ground-singlet ones are parity-forbidden.^9,10^ Due to these symmetry considerations, the negative interaction between emitters, commonly termed as J-aggregate, leads to a red-shifted coupled emission (Figure 1a).^10^ Conversely, if the interaction is positive (*J*_C_ > 0, “head-to-head” interaction), the triplet excitonic state has a higher energy than the excited state of an individual emitter. Therefore, the positive interaction between emitters, known as H-aggregate, results in a spectral blue shift (Figure 1b).^10^

**Figure 1:**
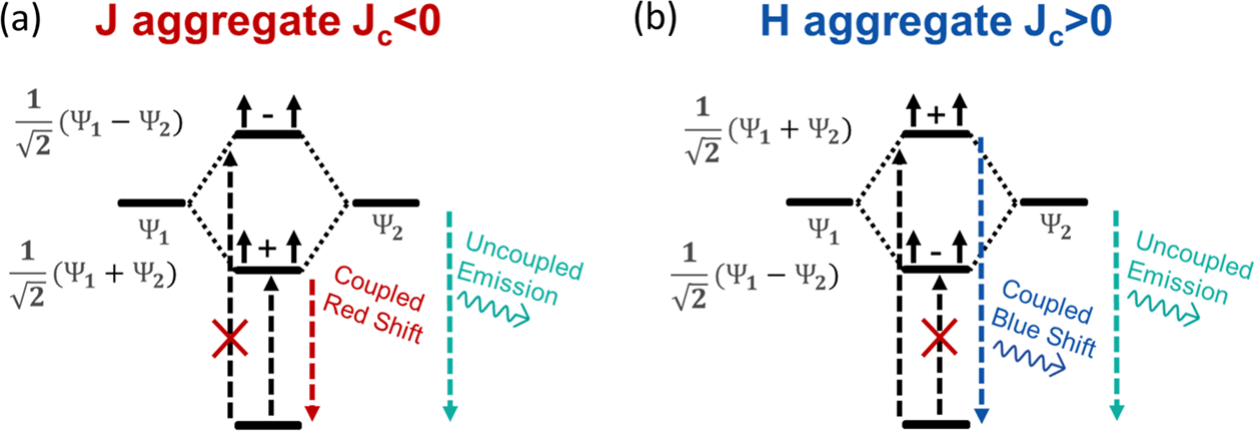
Underlying mechanism of dipole–dipole coupling-influenced collective emission behavior. (a, b) Energy level diagrams for the Coulomb dipole–dipole interaction between transition dipoles for a dimer of (a) “head-to-tail” interaction, J-aggregate, and (b) “head-to-head” interaction, H-aggregate. The transition between the ground and excited coupled states is allowed only to the state with aligned transition dipole moments. This bright state is the lower/upper coupled state for J/H-aggregates, respectively, leading to either a spectral red shift or blue shift in the coupled emission.

H/J-aggregate behavior is commonly found in aggregates of organic dye molecules^10−12^ and clusters of organic semiconductors.^13,14^ In these organic systems, the excitons are highly localized Frenkel excitons, positioned mainly between the electron-donating and electron-withdrawing groups.^10,15^ As a result, the type of interaction is mainly dictated by the relative orientation of the chromophores in the cluster. However, in many solid-state semiconductors, the excitons are delocalized Wainer–Mott excitons, which are free to move within the volume of the crystal.^16−18^ This theoretically opens the possibility of changing the collective interaction and emission characteristics without influencing the geometry of the cluster.

Ordered superlattice (SL) assemblies of nanocrystal quantum dots are promising platforms for such manipulation of the collective optical properties. The first observation of collective superfluorescent emission from CsPbBr_3_ nanocrystal superlattices was reported by Rainò et al.^5^ This collective emission was apparent only in ordered superlattices and measured at a cryogenic temperature of 5 K. This collective emission showed a distinct red shift with an accelerated emission rate relative to the emission of uncoupled nanocrystals. Several theoretical explanations for this collective accelerated emission were suggested, including Dicke superradiance and exciton delocalization.^5,19−21^ Following work showed collective emission from binary/ternary superlattices made from mixtures of CsPbBr_3_ and Fe_3_O_4_ or LaF_3_ nanocrystals, which arranged in many different structures.^22−25^ In these cases, the collective emission still originated from the coupling between the CsPbBr_3_ nanocrystals. Utilizing these collective optical properties, especially from superlattices of cesium lead halide perovskite nanocrystals, is promising for implementation in various applications ranging from bright and efficient emitters to high-energy ultrafast detectors.^26−28^ Therefore, the ability to control the type of coupling between nanocrystals and the resulting collective emission shown here is significant for these applications.

## Results

**Figure 2:**
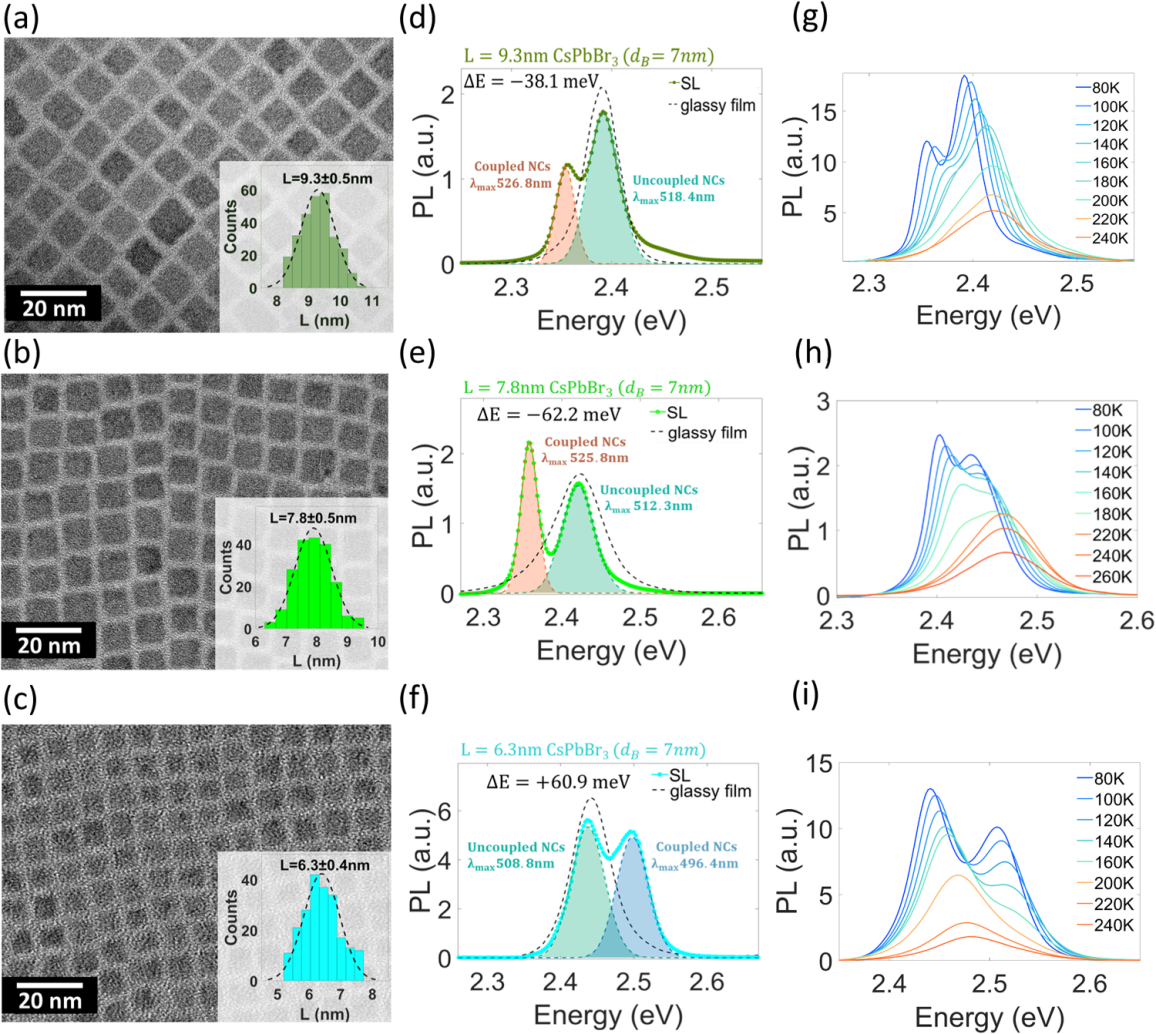
Quantum-confinement tunable collective emission of halide perovskite nanocrystals. (a–c) TEM micrographs and size distribution histograms of the nanocrystal building blocks for (a) 9.3 nm, (b) 7.8 nm, and (c) 6.3 nm cuboid-shaped CsPbBr_3_ nanocrystals. (d–f) PL spectrum at *T* = 80 K of superlattices made from (d) 9.3 nm, (e) 7.8 nm, and (f) 6.3 nm CsPbBr_3_ nanocrystals. The coupled emission is (d, e) red-shifted or (f) blue-shifted relative to the uncoupled emission present in PL from nanocrystal glassy films (black dashed lines). (g–i) PL spectra at different temperatures of superlattices made from (g) 9.3 nm, (h) 7.8 nm, and (i) 6.3 nm CsPbBr_3_ nanocrystals. In the larger nanocrystals, a red-shifted peak appears, and in smaller nanocrystals, a blue-shifted peak appears below a temperature of 180–200 K.

Formation of nanocrystal superlattices requires highly monodispersed and uniform nanocrystals.^29^ We synthesized monodispersed colloidal CsPbBr_3_ nanocrystals using the procedure reported by Dong et al.^30^ Modifying the reaction temperature and the Pb to halide precursor ratio allows size tunability of the nanocrystals (Figure 2a–c). Monodispersed CsPbBr_3_ nanocrystals with average edge sizes of ⟨*L*⟩ = 9.3 ± 0.5 nm, ⟨*L*⟩ = 7.8 ± 0.5 nm, and ⟨*L*⟩ = 6.3 ± 0.4 nm were obtained. Deposited nanocrystals self-assembled on the substrate, creating three-dimensional cubic-packed rectangular superlattices, along with nonassembled areas between the superlattices acting as colloidal glassy films (Figure S1). To probe the collective light emission, micro-photoluminescence (PL) measurements were conducted at cryogenic liquid nitrogen temperature (*T* = 80 K). All of the measured superlattices showed two distinct emission peaks at 80 K (Figure 2d–f). We identify one of these emission peaks as the spontaneous emission of individual uncoupled nanocrystals and the other as the cooperative emission of coupled nanocrystal systems. This determination is based on the PL of unorganized areas and on temperature-dependent measurements.

Unlike the two PL peaks in superlattices, spectra of unorganized glassy film areas at 80 K had only one PL peak (black dashed lines), which coincided with one of the two PL peaks of the superlattices. Therefore, we assign this peak to the spontaneous emission of uncoupled nanocrystals. The additional PL peak in superlattices is of coupled nanocrystal systems, which require the high structural order found in the superlattices. Different superlattices showed different ratios between the emission peaks, likely due to defects in the superlattices^31^ and reduced structural order required for the collective emission.

An additional method for identifying the coupled emission is by the temperature evolution of the PL spectra, as shown in Figure 2g–i. PL spectra of superlattices at a temperature range of 200 K < *T* < 273 K showed only a single peak, which red-shifts during cooling due to crystallographic changes in the perovskite structure.^32−34^ Below a threshold temperature of *T* < 180–200 K, a second emission peak formed. As coupling between excited nanocrystals requires interaction between transition dipoles, thermal fluctuations at higher temperatures hinder this process.^35,36^ The collective emission was reversible with the temperature changes, as heating above the threshold temperature made it disappear and cooling it below the threshold temperature again made it reappear. This indicates that the additional peak is not the result of irreversible changes in the system. Moreover, the intensity of the collective emission increases greatly upon a decrease in temperature, more than the intensity increment of the uncoupled nanocrystals. This is likely because reducing the temperature increases the probability of coupling between nanocrystals and, consequently, enhances the collective emission. Therefore, the emission peak at higher temperatures is again attributed to uncoupled nanocrystals, whereas the additional peak formed at lower temperatures (only in superlattices) is attributed to coupled nanocrystals.

Red-shifted coupled emission at cryogenic temperatures was previously reported in the CsPbBr_3_ superlattice as superfluorescent emission.^5^ In our measurements, however, the collective emission showed different spectral characteristics depending on the nanocrystal building blocks. In the cases of 9.3 and 7.8 nm nanocrystals, the coupled emission is red-shifted relative to the uncoupled peak. In contrast, superlattices made of smaller 6.3 nm CsPbBr_3_ nanocrystals showed drastically different coupled PL at temperatures of *T* ≤ 180 K. The coupled nanocrystal emission in this case was blue-shifted relative to the uncoupled emission. In addition to the red shift, we also observe spectral narrowing as shown in Figure 2d,e. This is a known property of superfluorescent J-aggregates where the coupled emission is narrower by a factor of √*N* due to exchange narrowing.^37^ In the case of the blue-shifted coupled PL, we do not observe a spectral narrowing but comparable width or broadening as commonly shown in molecular H-aggregates.^10^ In terms of the exciton interaction theory discussed previously, superlattices of small CsPbBr_3_ nanocrystals behave similarly to H-aggregates, while superlattices of larger nanocrystals behave like J-aggregates.

**Figure 3:**
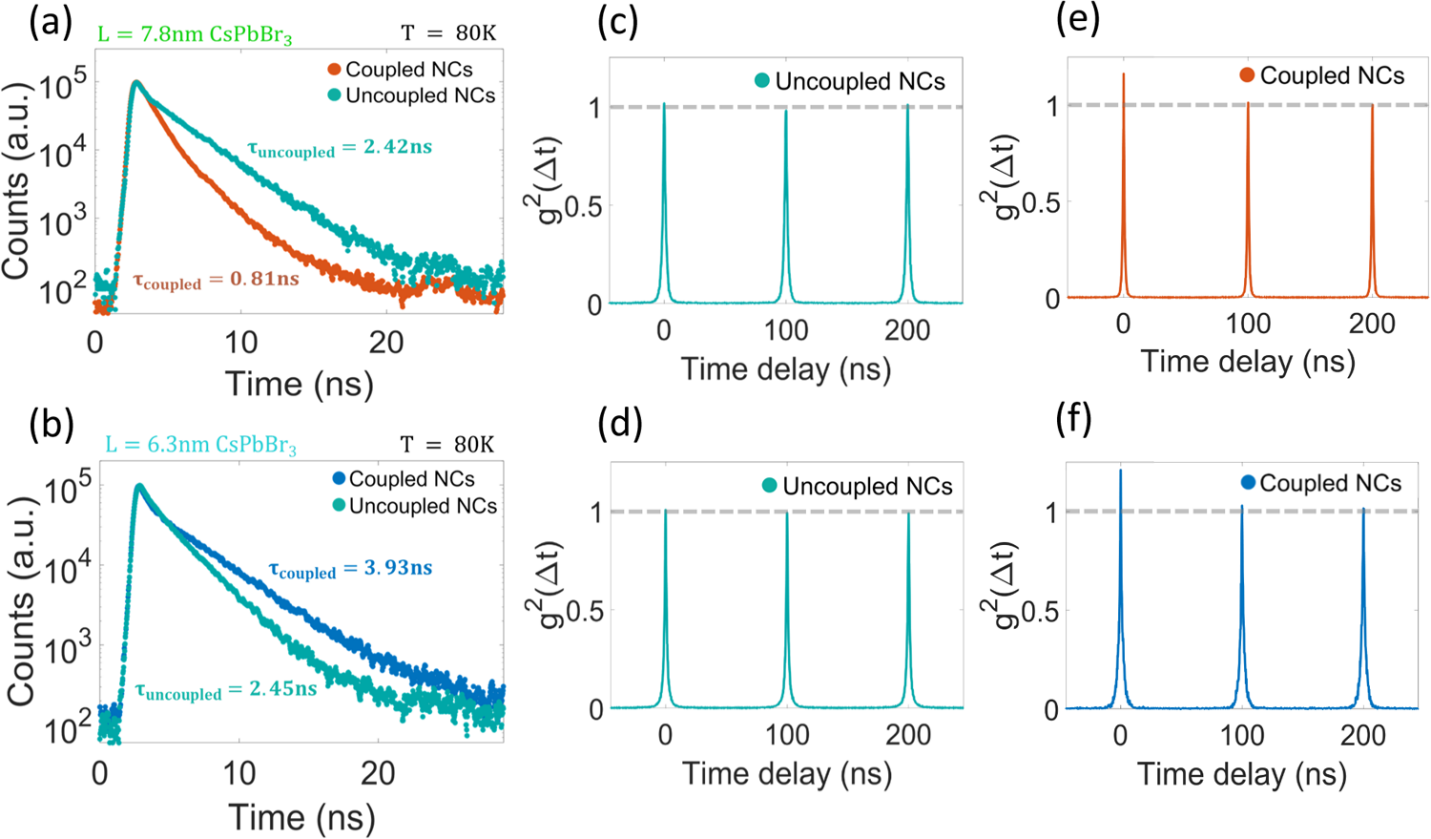
Temporal and correlative characteristics of the different collective emissions. (a, b) Time-resolved PL at 80 K of superlattices made from (a) 7.8 nm and (b) 6.3 nm CsPbBr_3_ nanocrystals. Average emission rates show (a) accelerated red-shifted coupled emission and (b) slower emission rate for the blue-shifted coupled emission. (c–f) Second-order correlation function measurements, obtained with a Hanbury–Brown and Twiss setup using pulsed excitation with 10 MHz repetition rate, for SLs made from (c, e) 7.8 nm and (d, f) 6.3 nm CsPbBr_3_ nanocrystals. Both red-shifted (e) and blue-shifted (f) collective emissions of coupled nanocrystals show photon bunching at a zero time delay, while emission of the uncoupled nanocrystals (c, d) shows Poissonian photon emission statistics.

In addition to the spectral changes, the emerging collective emissions showed different temporal and correlative behaviors compared to uncoupled nanocrystal emission depending on the type of coupling. In the case of collective red-shifted emission, the coupled emission has an accelerated decay rate relative to uncoupled nanocrystals, as shown by time-resolved photoluminescence (TRPL) in Figure 3a. As the collective transition dipole of a J-aggregate is the sum of the interacting transition dipoles, this emission is accelerated by a factor of the number of coupled emitters in the collective system *N*
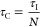
2where τ_C_ and τ_I_ are the radiative lifetimes for the coupled system of emitters and individual uncoupled emitters, respectively. For this reason, J-aggregates are often characterized as having superfluorescent emission.^2,10,38^ In our case, the collective emission of superlattices made from 7.8 nm CsPbBr_3_ nanocrystals showed 2–3 times higher emission rate than that of uncoupled nanocrystals. According to eq 2, this indicates that the effective number of coupled emitters is 2–3 nanocrystals. This increase in emission rate is consistent with previously reported measurements of 2.7 times faster superfluorescent collective emission rate in CsPbBr_3_ superlattices.^5^ On the other hand, collective blue-shifted emission showed a slower emission rate than the uncoupled nanocrystals, as shown in Figure 3b. This is a known property of H-aggregates, as this type of coupling introduces a competing nonradiative decay process toward the dark state with opposite transition dipoles.^10,39^ Despite the longer lifetime in the collective blue-shifted emission, we do not attribute it to the subradiant behavior, in which the emission rate is slower with the increase of the number of coupled emitters in the system.

We additionally measured the emission statistics of coupled and uncoupled nanocrystals using Hanbury–Brown and Twiss setups with pulsed excitation, to study the correlative properties of both types of coupling. Figure 3c–f shows the second-order correlation function (*g*^2^(Δ*t*)) of the emitted light. Uncoupled emission from both samples showed a Poissonian distribution of photon arrival times, with *g*^2^(0) = 1 (Figure 3c,d). On the other hand, collective emission from both types of coupling showed pronounced photon bunching *g*^2^(0) > 1, indicating a process of multiphoton emission bursts originating from the couplings (Figure 3e,f).^5^ Second-order correlation function values from longer time ranges, accounting for the integral intensity of more excitation pulses, are shown in Figure S4. The 7.8 nm CsPbBr_3_ nanocrystals showed *g*^2^(0) = 1.21 ± 0.02, and the 6.3 nm sample, despite its slower collective emission rate, showed *g*^2^(0) = 1.24 ± 0.01. These bunching results are 9 and 16 times the standard deviation above average, respectively. This indicates that despite the differences in spectral and temporal characteristics, both types of coupling resulted in correlative multiphoton emission.

We emphasize that despite the shared similarities between nanocrystal superlattices and molecular H/J-aggregates, shown by the collective energy shifts and the time-resolved behaviors, there are also striking differences. For example, some systems of interacting emitters, including molecular aggregates, were measured as having antibunching emission statistics.^40^ However, these results are different from our observations as an emission from these entangled superradiant states is in the single-photon superradiance regime, meaning that even when a single photon is emitted, the radiative rate is enhanced due to the relaxation being from a symmetric delocalized state with an enhanced oscillator strength.^21,41^ Our observed photon bunching from perovskite nanocrystal superlattices, along with previous reports,^5^ suggest interactions in the multiexcitation regime, resulting in multiphoton correlative emission.

In explaining the difference in collective emission between superlattices of large and small nanocrystals, we considered two options. One option is that a change in the nanocrystal size leads to a difference in the nanocrystal packing, thereby changing the conformation between neighboring transition dipoles. Recent work indeed showed that CsPbBr_3_ nanocrystal superlattice packing is affected by changes in the nanocrystal size and colloidal softness.^42,43^ The second option is that coupling differences arise from the effect of quantum confinement. To determine which mechanism is more likely, we changed the extent of quantum confinement without significantly changing the nanocrystal size and hence the related nanocrystal packing. This was achieved via a room-temperature anion exchange method, which in halide perovskite nanocrystals is facile, topotactic, and greatly affecting the Bohr diameter of excitons in the nanocrystals.^44,45^ The anion exchange process was conducted on the 6.3 and 7.8 nm CsPbBr_3_ nanocrystals reported previously, to obtain mixed-halide nanocrystals with different amounts of iodide or chloride. This effectively changes the composition of the nanocrystal and the extent of quantum confinement without significant changes to the physical size or colloidal softness of the nanocrystals.^44^ The extent of quantum confinement *x* may be defined as
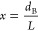
3where *d*_B_ is the Bohr diameter of excitons in the nanocrystal and *L* is the nanocrystal size. The Bohr diameters of CsPbCl_3_/CsPbBr_3_/CsPbI_3_ are 4 nm/7 nm/12 nm, respectively reflecting the difference in the dielectric function of the perovskite matrix and the effective mass of charge carriers.^46^ In the mixed-halide nanocrystals, the Bohr diameter was determined by the weighted mean of the pure compositions. Anion exchange of Br^–^ to I^–^ increases the Bohr diameter and the extent of quantum confinement. Alternatively, Br^–^ to Cl^–^ exchange reduces the Bohr diameter and therefore effectively decreases the extent of quantum confinement.

**Figure 4:**
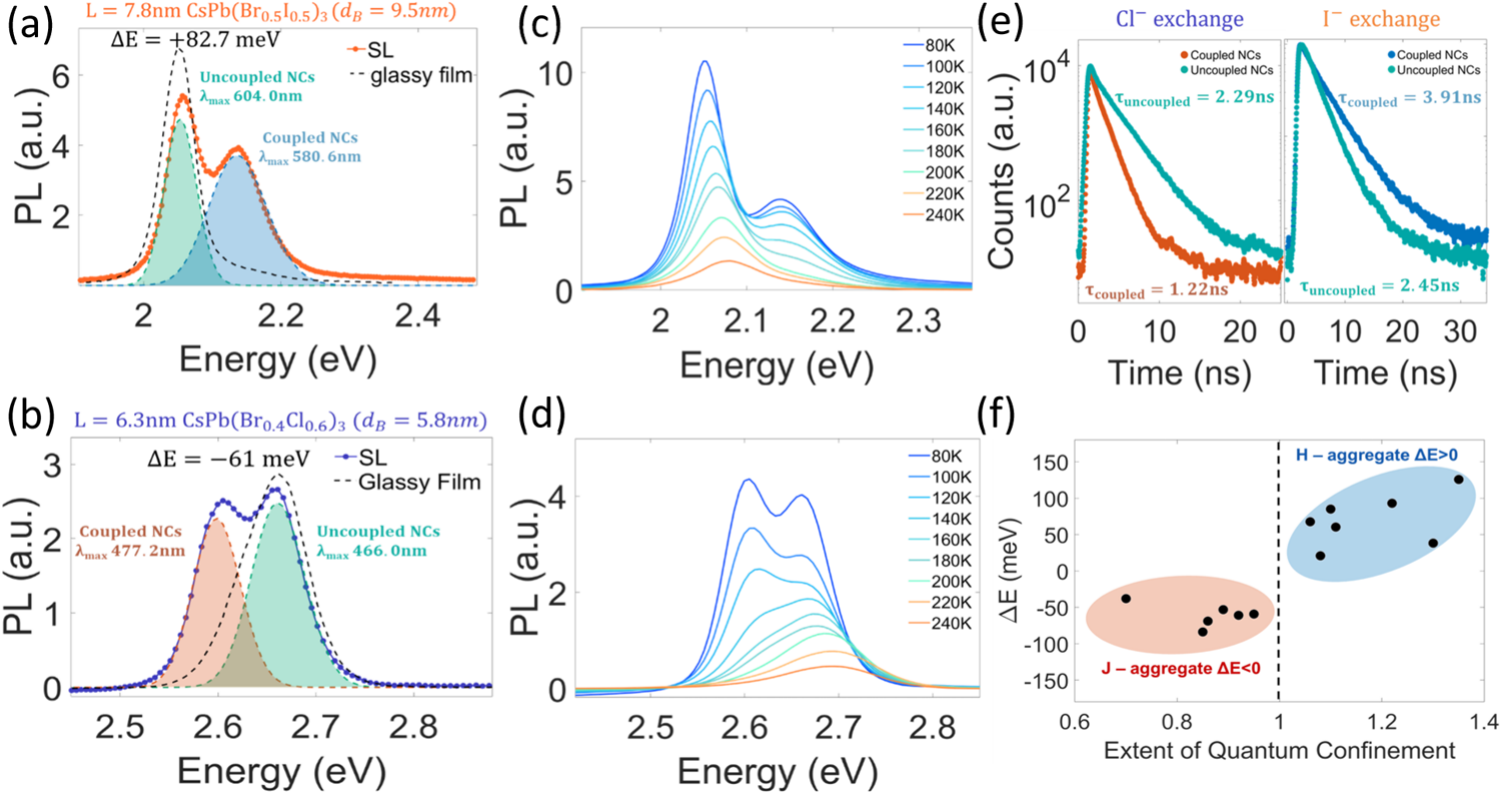
Composition control over quantum confinement and the resulting collective emission. (a, b) PL spectrum at *T* = 80 K of superlattices made of anion-exchanged (a) 7.8 nm CsPb(Br_0.5_I_0.5_)_3_ and (b) 6.3 nm CsPb(Br_0.4_Cl_0.6_)_3_ nanocrystals. The compositional changes modify the extent of quantum confinement and switch the collective spectral behaviors shown previously. (c, d) PL spectra at different temperatures of superlattices made of (c) 7.8 nm CsPb(Br_0.5_I_0.5_)_3_ and (d) 6.3 nm CsPb(Br_0.4_Cl_0.6_)_3_ nanocrystals. In chloride-exchanged nanocrystals, a red-shifted peak appears, and in iodide-exchanged nanocrystals, a blue-shifted peak appears at cryogenic temperatures. (e) Time-resolved PL at 80 K of superlattice made of (right) 7.8 nm CsPb(Br_0.5_I_0.5_)_3_ and (left) 6.3 nm CsPb(Br_0.4_Cl_0.6_)_3_. (f) Measured coupled PL energy difference vs the extent of quantum confinement of the nanocrystal building blocks.

Composition control over quantum confinement did change the collective spectral and temporal behaviors. Superlattices made of iodide-exchanged 7.8 nm CsPb(Br_0.5_I_0.5_)_3_ nanocrystals, with an effective Bohr diameter of 9.5 nm, changed their previously red-shifted J-aggregate-like collective emission behavior and showed a coupled blue-shifted H-aggregate-like behavior as a result of the quantum confinement (Figure 4a,c). The same spectral behavior was observed in other confined iodide-exchanged 7.8 nm CsPb(Br_0.7_I_0.3_)_3_ and CsPb(Br_0.3_I_0.7_)_3_ nanocrystals with effective Bohr diameters of 8.6 nm and 10.5 nm, respectively (Figure S8). In addition, superlattices made of chloride-exchanged 6.3 nm CsPb(Br_0.4_Cl_0.6_)_3_ nanocrystals, with an effective Bohr diameter of 5.8 nm, changed their previous collective emission from blue-shifted H-aggregate-like to a red-shifted J-aggregate-like behavior as a result of the weaker quantum confinement (Figure 4b,d).

In addition to the spectral changes in collective emission, the emission rates were modified by the anion exchange, as well, shown by TRPL in Figure 4e. Coupled PL of the iodide-exchanged nanocrystals showed a slower emission rate, while chloride-exchanged nanocrystals showed an accelerated emission by a factor of 2 relative to the uncoupled emission. Figure 4f presents the energy difference between coupled and uncoupled emissions vs the extent of quantum confinement from 13 samples with nanocrystals of various compositions and sizes. In all nanocrystals with *x* > 1, the collective emission behavior was blue-shifted and behave similarly to the H-aggregate, while all nanocrystals with *x* < 1 showed red-shifted J-aggregate-like collective emission.

**Figure 5:**
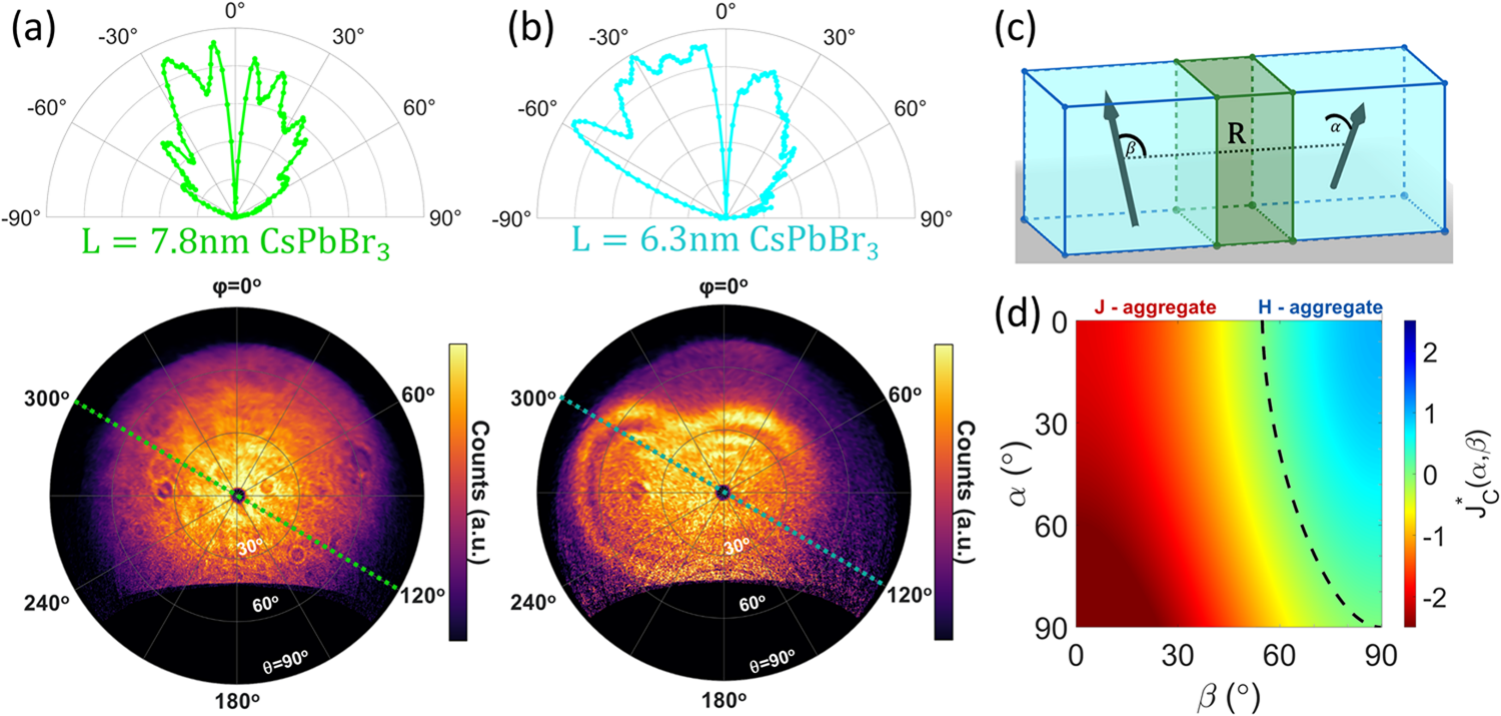
Quantum confinement effects on the transition dipole orientation and coupling type. (a, b) Angular resolved cathodoluminescence emission patterns measured from superlattices made of (a) 7.8 nm, and (b) 6.3 nm CsPbBr_3_ nanocrystals. The upper panels show intensity vs polar angle θ along the azimuthal axis of φ = 120°/300° marked in dashed lines on the 2D projections. (c) Schematic of the coupled nanocrystal dimer with a nonplanar transition dipole conformation. (d) Dipole–dipole coupling conformation factor calculation for nonplanar transition dipoles as a function of inclination angle β and angle between transition dipole planes α.

To better understand why quantum confinement changes the coupling behavior, we set out to measure the alignment of the transition dipoles of the nanocrystals. In excitonic luminescent materials, light emission has a cosine angular distribution with a peak oriented perpendicular to the transition dipole moment.^47^ Due to this angular distribution, it is possible to understand the orientation of transition dipoles in materials from angular-dependent emission patterns. This method was previously used to determine the transition dipole orientation in molecules,^48^ layered nanomaterials,^49^ CdSe nanoplates,^50^ and CsPbBr_3_ nanocrystals.^51^ We measured angular-dependent emission patterns locally from 2 to 3 columns of nanocrystals in the superlattice using cathodoluminescence (CL) scanning electron microscopy.^42,52^ The angular resolved data were analyzed and plotted as polar maps shown in Figure 5a,b, with each point corresponding to a different polar angle (θ) and azimuthal angle (φ).^48^

Emission from 7.8 nm CsPbBr_3_ nanocrystal superlattices was isotropic along φ, with most of the emission (80.3%) directed to low polar angles in the range of θ = 5–45°. The 6.3 nm sample showed different angular-dependent emission patterns with preferred azimuthal angles. The preferred azimuthal angles were different for different superlattices in this sample as shown in Figure S9. The 6.3 nm nanocrystals showed a polar angle directionality of θ = 5–45° (57.9% of the emitted light) as well but with an additional dominant emission (40.3%) at higher polar angles of up to 68°. These differences in angular distribution are assigned to changes in the preferred transition dipole orientation in the nanocrystals, which is affected by the size of the nanocrystals via quantum confinement. This is further supported by previous observations of transition dipole-preferred alignment changes due to confinement in CsPbBr_3_ nanocrystals.^51,53^

The introduced preferred transition dipole orientation in smaller nanocrystals leads to a different transition dipole conformation between neighboring nanocrystals and therefore dictates the coupling type (Figure 5c,d). If the transition dipole size (|**μ**|) is the same for both coupled emitters in a dimer, the dipole–dipole coupling energy derived from eq 1 is
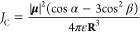
4where β is the angle between **μ** and **R⃗** and α is the angle between transition dipoles. In effect, the dipole–dipole interaction sign is governed by β and α angle-dependent conformation factors (*J*_C_^*^). Below a critical inclination angle β_C_ (54.7° for α = 0°), *J*_C_ < 0 i.e., red-shift coupling. Meanwhile, for the transition dipole conformation with higher than the critical inclination angle, *J*_C_ > 0 i.e., blue-shift coupling. From eq 4, the critical inclination angle is given by
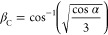
5Our measured transition dipole conformation agrees with the spectral behaviors we observed (Figure 2e,f). In the 7.8 nm nanocrystals, we expect α = 0° due to the isotropic pattern in φ, from the emission directionality to small polar angles in θ we find a β of 54.7°, and therefore, the expected coupling behavior is indeed a red-shifted-like J-aggregate as was observed. In the 6.3 nm case, the measured high-polar angle θ emission component indicates that β > β_C_(α). Therefore, the extracted conformation of transition dipoles in this sample is expected to cause a blue-shift H-aggregate-like coupling as was measured.

## Conclusions

We report switching the collective optical behavior of perovskite nanocrystal superlattices between two distinct regimes. Merging Kasha exciton interaction theory with the Dicke superradiance model provides a better understanding of exciton interactions and collective emission phenomena at the solid state. Using this understanding, we assign a key role for dipole–dipole interactions in the collective emission process that is tuned through control over the quantum confinement. Weaker confined nanocrystals behave like J-aggregates with a coupled emission red shift and an accelerated emission rate, while stronger confined nanocrystals behave like H-aggregates with a blue-shifted coupled emission and a reduced emission rate. Switching between these behaviors is possible by changing the nanocrystal size and halide composition. The demonstrated ability to tune collective light emission is especially important for future implementation of these nanomaterials in optoelectronic applications utilizing their emerging collective properties.

## Methods

### Materials

Cesium carbonate (99.9%, Aldrich), hexane (A.R., Aldrich), lead bromide (99.998%, Aesar), lead chloride (99.999%, Aldrich), lead iodide (99%, Aldrich), octadecene (90%, Acros), oleic acid (90%, Aldrich), oleylamine (98%, Aldrich), toluene (99.8%, Aldrich), and zinc bromide (99.9%, Aesar) were used.

All chemicals were used as purchased without further purification.

### Synthesis of CsPbBr_3_ Nanocrystals, Anion Exchange, and Superlattice Preparation

First, the Cs-oleate precursor was prepared in a 50 mL three-necked round-bottomed flask by dissolving Cs_2_CO_3_ (0.25 g) in a mixture of oleic acid (0.8 g) and octadecene (7 g) at 150 °C for 10 min under a N_2_ atmosphere on a Schlenk line. The precursor solution of Pb and Br was prepared by dissolving PbBr_2_ (75 mg) and a varying amount of ZnBr_2_ in a mixture of octadecene (5 mL), oleic acid (2 mL), and oleylamine (2 mL) in a 25 mL three-necked round-bottomed flask under a N_2_ atmosphere at 120 °C for 10 min. After the temperature of the precursor solution of Pb and Br was set, 0.4 mL of Cs precursor solution was injected to initiate the reaction. The reaction was quenched after a few seconds in an ice-water bath. The product was centrifuged twice at 9000 and 3500 rpm and dispersed in clean toluene to obtain monodispersed nanocrystals. The anion exchange process was conducted under ambient conditions. Halide stock solution was made by mixing 5 mL of clean toluene with 0.1 mL of oleic acid and 100 mg of PbX_2_ salt. 1 mL of isolated nanocrystals in toluene was mixed with the desired amount of lead halide stock solution overnight to obtain mixed-halide nanocrystals. The SLs were prepared by a drop casting process of the nanocrystal solution in toluene on the desired substrate. Silicon wafer was used as a substrate for SEM and PL characterization and carbon film on 300 mesh copper grids for TEM characterization. 25 μL volume was cast and dried under ambient conditions for several hours (usually 3–5 h) followed by overnight vacuuming.

### Liquid Nitrogen Micro-photoluminescence (PL), Time-Resolved Photoluminescence (TRPL), and Photon Statistics Measurements

Spectroscopic characterizations were performed using an Edinburgh FLS1000 spectrometer coupled to a Nikon Eclipse UPRIGHT microscope with a THMS350 V Linkam temperature-controlled vacuum cryogenic stage with LNP95. All the deposited nanocrystal samples were loaded into the cryostat and vacuumed to prevent ice formation; during temperature changes, the system had 180 s stabilization time. The samples were excited either by an Edinburgh Xe lamp or Edinburgh 375 or 405 nm efficient pulse laser (EPL). TRPL measurements were performed in a multichannel scaling (MCS) mode with a monochromator used for separating the coupled and uncoupled emissions. Photon statistics and second-order correlation function measurements were conducted on the same setup using a 405 nm EPL with an appropriate band-pass filter and two avalanche photodiodes (Micro Photon Devices, 100 μm PDM SPAD) and 50:50 beamsplitter splitting the light between them.

### Transmission Electron Microscopy (TEM) Characterization

One drop of dilute nanocrystal solution in hexane (1:20 dilution) was cast onto a TEM grid (carbon film only on a 300 mesh copper grid). The samples were observed in TEM mode with a Thermo Fisher/FEI Tecnai G^2^ T20 S-Twin LaB_6_ TEM operated at 200 K with a Gatan Rio9 CMOS camera.

### Scanning Electron Microscopy (SEM)

#### High-Resolution Scanning Electron Microscopy (SEM)

One drop of dilute nanocrystal solution was cast on a silicone substrate for SEM characterization using a Zeiss Ultra-Plus FEG-SEM. Samples were placed at a working distance of 3 mm and measured using acceleration voltages between 1 and 5 kV.

#### Angular Resolved Cathodoluminescence (CL) SEM

CL experiments were conducted on a Thermo Fisher Apreo 2S scanning electron microscope with a Delmic SPARC CL module equipped with a parabolic mirror. A beam energy of 10 keV was used with an electron probe current of 400 pA. The electron beam spot size was estimated to be around 3 nm at 10 keV. The CL emission is analyzed with a high-speed CDD camera (Andor Newton 920). For angular resolved emission measurements, the system was equipped with a band-pass filter of 500/50 nm, and the acquisition time was 5 s.

## Supporting Information Available

Additional PL measurements of the collective light emissions, excitation power dependence, additional CL mappings, and structural characterization of the superlattices (PDF)

## References

[ref1] Dicke R. H. Coherence in spontaneous radiation processes. Phys. Rev. 1954, 93, 99–110. 10.1103/PhysRev.93.99.

[ref2] Gross M.; Haroche S. Superradiance: an essay on the theory of collective spontaneous emission. Phys. Rep. 1982, 93, 301–396. 10.1016/0370-1573(82)90102-8.

[ref3] Bonifacio R.; Lugiato L. A. Cooperative radiation processes in two-level systems: Superfluorescence. Phys. Rev. A 1975, 12, 587 10.1103/PhysRevA.12.587.

[ref4] Skribanowitz N.; Herman P.; Macgillivray J. C.; Feld M. S. Observation of Dicke Superradiance in Optically Pumped HF Gas. Phys. Rev. Lett. 1973, 30, 309 10.1103/PhysRevLett.30.309.

[ref5] Rainò G.; Becker M. A.; Bodnarchuk M. I.; et al. Superfluorescence from lead halide perovskite quantum dot superlattices. Nature 2018, 563, 671–675. 10.1038/s41586-018-0683-0. 30405237

[ref6] Miyajima K.; Kagotani Y.; Saito S.; Ashida M.; Itoh T. Superfluorescent pulsed emission from biexcitons in an ensemble of semiconductor quantum dots. J. Phys.: Condens. Matter 2009, 21, 195802 10.1088/0953-8984/21/19/195802. 21825497

[ref7] Dai D. C.; Monkman A. P. Observation of superfluorescence from a quantum ensemble of coherent excitons in a ZnTe crystal: Evidence for spontaneous Bose–Einstein condensation of excitons. Phys. Rev. B 2011, 84, 115206 10.1103/PhysRevB.84.115206.

[ref8] Mcrae E. G.; Kasha M. Enhancement of phosphorescence ability upon aggregation of dye molecules. J. Chem. Phys. 1958, 28, 721–722. 10.1063/1.1744225.

[ref9] Kasha M.; Rawls H. R.; El-Bayoumi M. A. The Exciton Model In Molecular Spectroscopy. Pure Appl. Chem. 1965, 11, 371–392. 10.1351/pac196511030371.

[ref10] Hestand N. J.; Spano F. C. Expanded Theory of H- and J-Molecular Aggregates: The Effects of Vibronic Coupling and Intermolecular Charge Transfer. Chem. Rev. 2018, 118, 7069–7163. 10.1021/acs.chemrev.7b00581. 29664617

[ref11] Spano F. C. The spectral signatures of frenkel polarons in H- And J-aggregates. Acc. Chem. Res. 2010, 43, 429–439. 10.1021/ar900233v. 20014774

[ref12] Wu Q.; Zhang T.; Peng Q.; Wang D.; Shuai Z. Aggregation induced blue-shifted emission-the molecular picture from a QM/MM study. Phys. Chem. Chem. Phys. 2014, 16, 5545–5552. 10.1039/C3CP54910K. 24509542

[ref13] Eder T.; Stangl T.; Gmelch M.; et al. Switching between H- and J-type electronic coupling in single conjugated polymer aggregates. Nat. Commun. 2017, 8, 1641 10.1038/s41467-017-01773-0. 29158508 PMC5696370

[ref14] Sarkar T.; Schneider S. A.; Ankonina G.; et al. Tuning Intra and Intermolecular Interactions for Balanced Hole and Electron Transport in Semiconducting Polymers. Chem. Mater. 2020, 32, 7338–7346. 10.1021/acs.chemmater.0c02199.

[ref15] Bardeen C. J. The structure and dynamics of molecular excitons. Annu. Rev. Phys. Chem. 2014, 65, 127–148. 10.1146/annurev-physchem-040513-103654. 24313684

[ref16] Tamarat P.; Bodnarchuk M. I.; Trebbia J. B.; et al. The ground exciton state of formamidinium lead bromide perovskite nanocrystals is a singlet dark state. Nat. Mater. 2019, 18, 717–724. 10.1038/s41563-019-0364-x. 31086320

[ref17] Akkerman Q. A.; Rainò G.; Kovalenko M. V.; Manna L. Genesis, challenges and opportunities for colloidal lead halide perovskite nanocrystals. Nat. Mater. 2018, 17, 394–405. 10.1038/s41563-018-0018-4. 29459748

[ref18] Chiao Z. Y.; Chen Y. C.; Chen J. W.; et al. Full-color generation enabled by refractory plasmonic crystals. Nanophotonics 2022, 11, 2891–2899. 10.1515/nanoph-2022-0071. 39634090 PMC11501569

[ref19] Blach D. D.; Lumsargis V. A.; Clark D. E.; et al. Superradiance and Exciton Delocalization in Perovskite Quantum Dot Superlattices. Nano Lett. 2022, 22, 7811–7818. 10.1021/acs.nanolett.2c02427. 36130299

[ref20] Masson S. J.; Asenjo-Garcia A. Universality of Dicke superradiance in arrays of quantum emitters. Nat. Commun. 2022, 13, 2285 10.1038/s41467-022-29805-4. 35477714 PMC9046277

[ref21] Zhu C.; Boehme S. C.; Feld L. G.; et al. Single-photon superradiance in individual caesium lead halide quantum dots. Nature 2024, 626, 535 10.1038/s41586-023-07001-8. 38297126 PMC10866711

[ref22] Cherniukh I.; Rainò G.; Stöferle T.; et al. Perovskite-type superlattices from lead halide perovskite nanocubes. Nature 2021, 593, 535–542. 10.1038/s41586-021-03492-5. 34040208

[ref23] Cherniukh I.; Rainò G.; Sekh T. V.; et al. Shape-Directed Co-Assembly of Lead Halide Perovskite Nanocubes with Dielectric Nanodisks into Binary Nanocrystal Superlattices. ACS Nano 2021, 15, 16488–16500. 10.1021/acsnano.1c06047. 34549582 PMC8552496

[ref24] Cherniukh I.; Sekh T. V.; Rainò G.; et al. Structural Diversity in Multicomponent Nanocrystal Superlattices Comprising Lead Halide Perovskite Nanocubes. ACS Nano 2022, 16, 7210–7232. 10.1021/acsnano.1c10702. 35385663 PMC9134504

[ref25] Sekh T. V.; Cherniukh I.; Kobiyama E.; et al. All-Perovskite Multicomponent Nanocrystal Superlattices. ACS Nano 2024, 18, 8423–8436. 10.1021/acsnano.3c13062. 38446635 PMC10958606

[ref26] Li X.; Liu X.; Liu X. Self-assembly of colloidal inorganic nanocrystals: Nanoscale forces, emergent properties and applications. Chem. Soc. Rev. 2021, 50, 2074–2101. 10.1039/D0CS00436G. 33325927

[ref27] Tang Y.; Poonia D.; van der Laan M.; et al. Electronic Coupling of Highly Ordered Perovskite Nanocrystals in Supercrystals. ACS Appl. Energy Mater. 2022, 5, 5415–5422. 10.1021/acsaem.1c03276. 35647492 PMC9131308

[ref28] Brennan M. C.; Toso S.; Pavlovetc I. M.; et al. Superlattices are greener on the other side: How light transforms self-assembled mixed halide perovskite nanocrystals. ACS Energy Lett. 2020, 5, 1465–1473. 10.1021/acsenergylett.0c00630.

[ref29] Auer S.; Frenkel D. Suppression of crystal nucleation in polydisperse colloids due to increase of the surface free energy. Nature 2001, 413, 711–713. 10.1038/35099513. 11607025

[ref30] Dong Y.; Qiao T.; Kim D.; et al. Precise Control of Quantum Confinement in Cesium Lead Halide Perovskite Quantum Dots via Thermodynamic Equilibrium. Nano Lett. 2018, 18, 3716–3722. 10.1021/acs.nanolett.8b00861. 29727576

[ref31] Clark D. E.; Lumsargis V. A.; Blach D. D.; et al. Quantifying Structural Heterogeneity in Individual CsPbBr _3_ Quantum Dot Superlattices. Chem. Mater. 2022, 34, 10200–10207. 10.1021/acs.chemmater.2c03153.

[ref32] Bertolotti F.; Protesescu L.; Kovalenko M. V.; et al. Coherent Nanotwins and Dynamic Disorder in Cesium Lead Halide Perovskite Nanocrystals. ACS Nano 2017, 11, 3819–3831. 10.1021/acsnano.7b00017. 28394579 PMC5800404

[ref33] Strandell D. P.; Kambhampati P. The Temperature Dependence of the Photoluminescence of CsPbBr3 Nanocrystals Reveals Phase Transitions and Homogeneous Linewidths. J. Phys. Chem. C 2021, 125, 27504–27508. 10.1021/acs.jpcc.1c09501.

[ref34] Shcherbakov-Wu W.; Sercel P. C.; Krieg F.; Kovalenko M. V.; Tisdale W. A. Temperature-Independent Dielectric Constant in CsPbBr3Nanocrystals Revealed by Linear Absorption Spectroscopy. J. Phys. Chem. Lett. 2021, 12, 8088–8095. 10.1021/acs.jpclett.1c01822. 34406780

[ref35] Mattiotti F.; Kuno M.; Borgonovi F.; Jankó B.; Celardo G. L. Thermal Decoherence of Superradiance in Lead Halide Perovskite Nanocrystal Superlattices. Nano Lett. 2020, 20, 7382–7388. 10.1021/acs.nanolett.0c02784. 32969667

[ref36] Adl H. P.; Gorji S.; Muñoz-Matutano G.; et al. Superradiance Emission and Its Thermal Decoherence in Lead Halide Perovskites Superlattices. Adv. Opt. Mater. 2023, 11, 2202497 10.1002/adom.202202497.

[ref37] Knapp E. W. Lineshapes of molecular aggregates, exchange narrowing and intersite correlation. Chem. Phys. 1984, 85, 73–82. 10.1016/S0301-0104(84)85174-5.

[ref38] Fidder H.; Knoester J.; Wiersma D. A. Superradiant emission and optical dephasing in J-aggregates. Chem. Phys. Lett. 1990, 171, 529–536. 10.1016/0009-2614(90)85258-E.

[ref39] Chaudhuri D.; Li D.; Che Y.; et al. Enhancing long-range exciton guiding in molecular nanowires by H-aggregation lifetime engineering. Nano Lett. 2011, 11, 488–492. 10.1021/nl1033039. 21175215

[ref40] Lange C. M.; Daggett E.; Walther V.; Huang L.; Hood J. D. Superradiant and subradiant states in lifetime-limited organic molecules through laser-induced tuning. Nat. Phys. 2024, 20, 836–842. 10.1038/s41567-024-02404-4.

[ref41] Trebbia J. B.; Deplano Q.; Tamarat P.; Lounis B. Tailoring the superradiant and subradiant nature of two coherently coupled quantum emitters. Nat. Commun. 2022, 13, 2962 10.1038/s41467-022-30672-2. 35618729 PMC9135760

[ref42] Levy S.; Be’er O.; Veber N.; Monachon C.; Bekenstein Y. Tuning the Colloidal Softness of CsPbBr 3 Nanocrystals for Homogeneous Superlattices. Nano Lett. 2023, 23, 7129–7134. 10.1021/acs.nanolett.3c02023. 37470186

[ref43] Boehme S. C.; Bodnarchuk M. I.; Burian M.; et al. Strongly Confined CsPbBr _3_ Quantum Dots as Quantum Emitters and Building Blocks for Rhombic Superlattices. ACS Nano 2023, 17, 2089–2100. 10.1021/acsnano.2c07677. 36719353 PMC9933619

[ref44] Akkerman Q. A.; D’Innocenzo V.; Accornero S.; et al. Tuning the optical properties of cesium lead halide perovskite nanocrystals by anion exchange reactions. J. Am. Chem. Soc. 2015, 137, 10276–10281. 10.1021/jacs.5b05602. 26214734 PMC4543997

[ref45] Nedelcu G.; Protesescu L.; Yakunin S.; et al. Fast Anion-Exchange in Highly Luminescent Nanocrystals of Cesium Lead Halide Perovskites (CsPbX3, X = Cl, Br, I). Nano Lett. 2015, 15, 5635–5640. 10.1021/acs.nanolett.5b02404. 26207728 PMC4538456

[ref46] Protesescu L.; Yakunin S.; Bodnarchuk M. I.; et al. Nanocrystals of Cesium Lead Halide Perovskites (CsPbX 3, X = Cl, Br, and I): Novel Optoelectronic Materials Showing Bright Emission with Wide Color Gamut. Nano Lett. 2015, 15, 2023 10.1021/nl5048779. PMC4462997 25633588

[ref47] Turro N. J. M. Molecular Photochemistry; University Science Books, 1991.

[ref48] Lieb M. A.; Novotny L.; Zavislan J. M. Single-molecule orientations determined by direct emission pattern imaging. J. Opt. Soc. Am. B 2004, 21, 1210–1215. 10.1364/JOSAB.21.001210.

[ref49] Schuller J. A.; Karaveli S.; Schiros T.; et al. Orientation of luminescent excitons in layered nanomaterials. Nat. Nanotechnol. 2013, 8, 271–276. 10.1038/nnano.2013.20. 23455984

[ref50] Gao Y.; Weidman M. C.; Tisdale W. A. CdSe Nanoplatelet Films with Controlled Orientation of their Transition Dipole Moment. Nano Lett. 2017, 17, 3837–3843. 10.1021/acs.nanolett.7b01237. 28534407

[ref51] Jurow M. J.; Morgenstern T.; Eisler C.; et al. Manipulating the Transition Dipole Moment of CsPbBr 3 Perovskite Nanocrystals for Superior Optical Properties. Nano Lett. 2019, 19, 2489–2496. 10.1021/acs.nanolett.9b00122. 30848600

[ref52] Coenen T.; Vesseur E. J. R.; Polman A. Angle-resolved cathodoluminescence spectroscopy. Appl. Phys. Lett. 2011, 99, 143103 10.1063/1.3644985. 21780758

[ref53] Becker M. A.; Vaxenburg R.; Nedelcu G.; et al. Bright triplet excitons in caesium lead halide perovskites. Nature 2018, 553, 189–193. 10.1038/nature25147. 29323292

