# Supplementary material for: Collective Interactions of Quantum-Confined Excitons in Halide Perovskite Nanocrystal Superlattices
Source: ACS Nano. 2024 Dec 26;19(1):963–71. doi: 10.1021/acsnano.4c12509 (PMC11921029; doi:10.1021/acsnano.4c12509)
Supplement: Supplementary file 1 — nn4c12509_si_001.pdf [file nn4c12509_si_001.pdf]

Supporting Information For:

Collective interactions of quantum confined excitons in halide perovskite nanocrystal superlattices

Shai Levy[1,2], Orr Be'er[1,2], Saar Shaek[1,2], Alexey Gorlach[2], Einav Scharf[3], Yonatan Ossia[3], Rotem Liran[3] Kobi Cohen[1,2], Rotem Strassberg[1,2], Ido Kaminer[2], Uri Banin[3], and Yehonadav Bekenstein[1,2]

1 Department of Materials Science and Engineering, Technion-Israel Institute of Technology, 32000 Haifa, Israel
2 The Solid-State Institute, Technion – Israel Institute of Technology, 32000 Haifa, Israel
3 Institute of Chemistry and the Center for Nanoscience and Nanotechnology, The Hebrew University of Jerusalem, 91904, Jerusalem, Israel

Additional Results

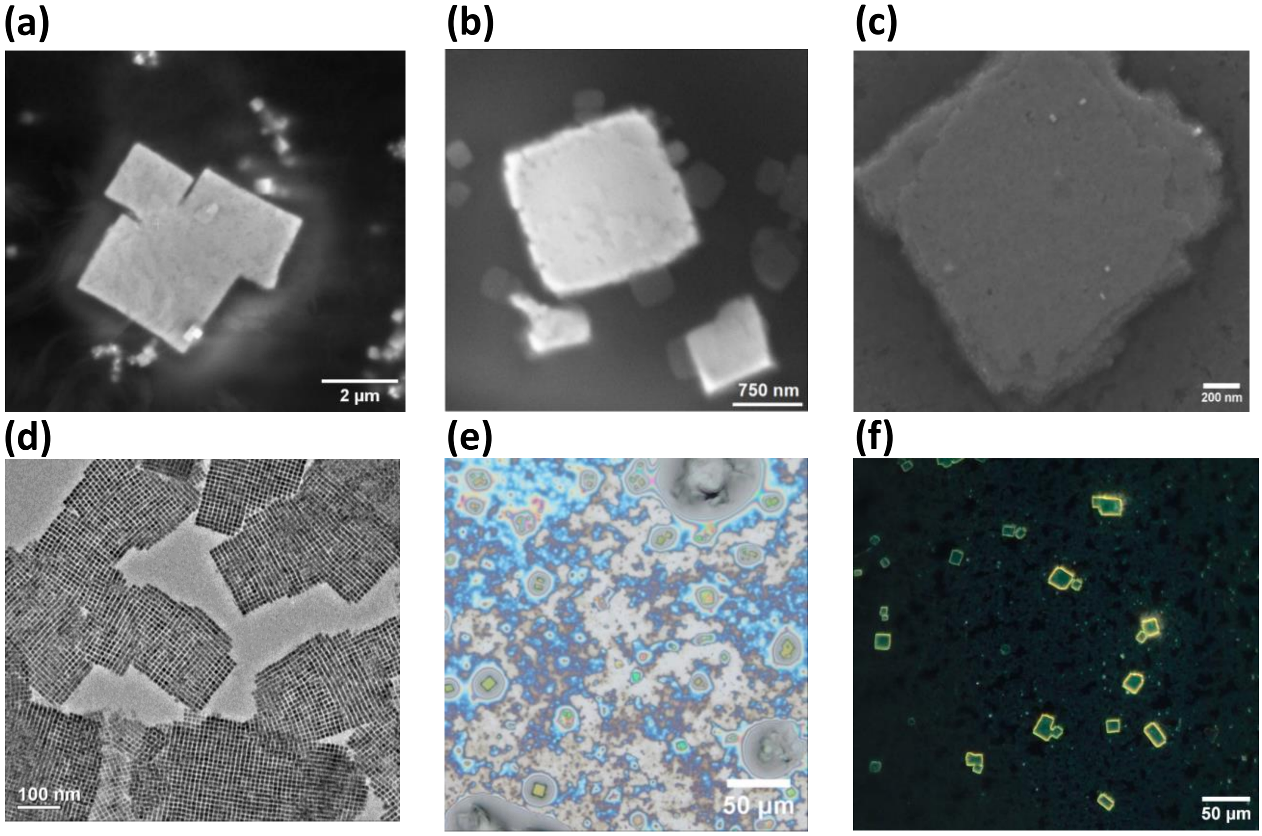

Figure S1. (a-c) SEM micrographs of rectangular $CsPbBr_3$ nanocrystal SLs, in (c) the surrounding colloidal glassy film is apparent. **(d)** Low magnification TEM micrograph of 7.8nm $CsPbBr_3$ nanocrystal SLs. **(e-f)** Optical microscope images of $CsPbBr_3$ nanocrystal SLs with surrounding colloidal glassy film; (e) under bright field (BF), and (f) under dark field (DF).

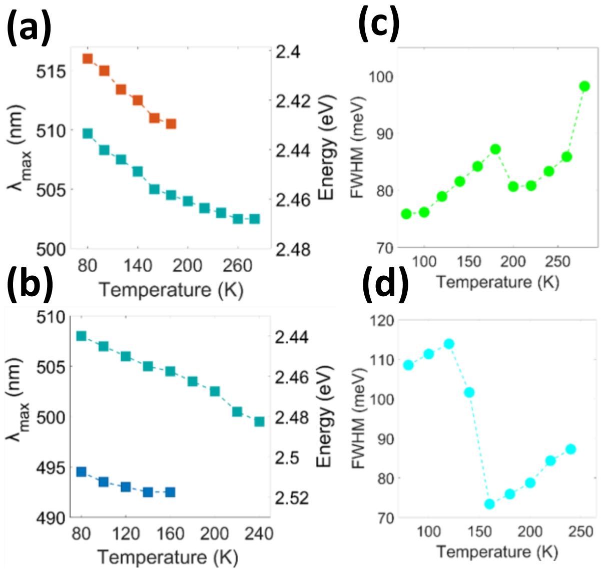

Figure S2. (a-b) peak emissions for coupled and uncoupled PL during temperature mappings of SLs made from (a) 7.8nm, and (b) 6.3nm $CsPbBr_3$ NCs. **(c-d)** Full width half maximum of PL spectra during temperature mappings of SLs made from (c) 7.8nm, and (d) 6.3nm $CsPbBr_3$ NCs. These plots show the coupled emission band forming below the critical temperature increasing the spectra width.

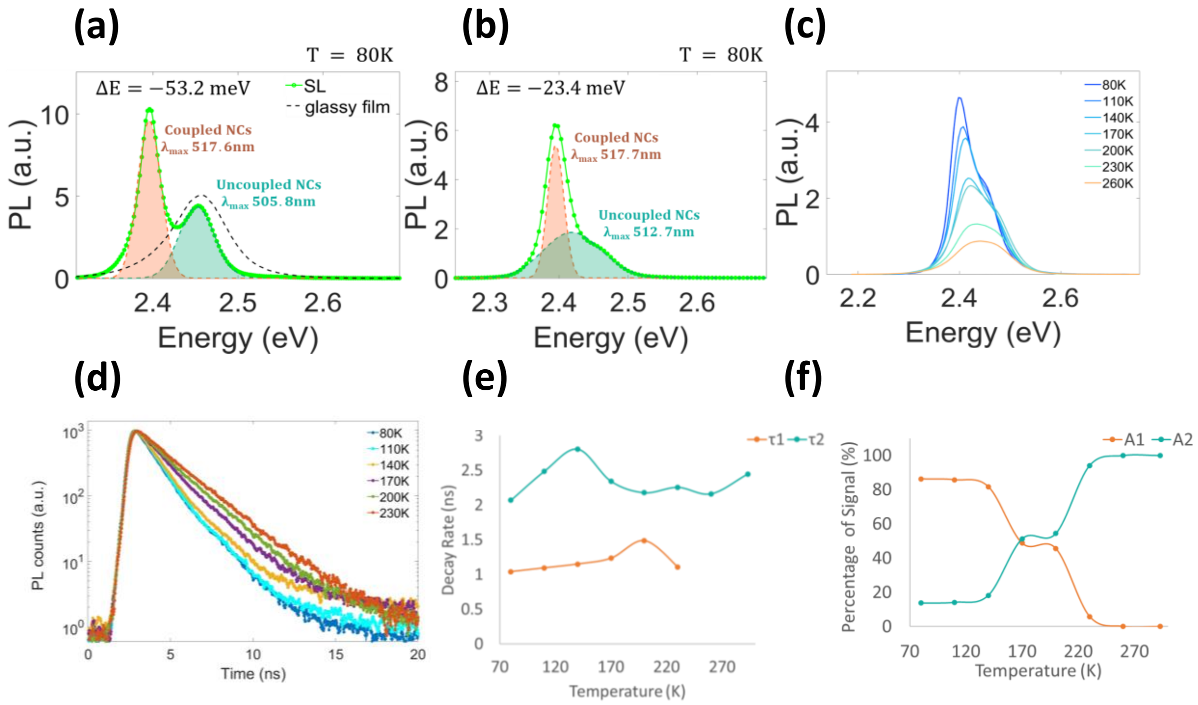

Figure S3. (a-b) Additional J aggregate behavior of weakly confined 7.8nm $CsPbBr_3$ nanocrystal SLs at 80K showing red-shifted PL of coupled NCs. **(c)** Temperature dependent PL spectra of (b). **(d)** TRPL at different temperatures in (c). The data matches biexponential decay for the combined coupled and uncoupled emissions. **(e-f)** Radiative lifetime (e), and percentage of emitted light (f) extracted from TRPL in panel (d). Below critical temp of ~200K the faster red-shifted emission appears and increases in overall intensity at lower temperatures.

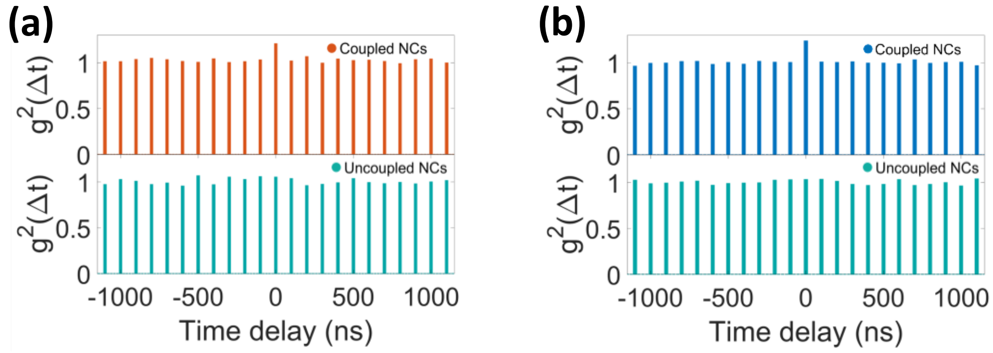

Figure S4. (a-b) Second-order correlation function measurements, obtained with a Hanbury–Brown and Twiss set-up using pulsed excitation with a 10 MHz repetition rate, and bin size of 20ns, for SLs made from (a) 7.8nm, and (b) 6.3nm CsPbBr3 NCs. Both red-shifted and blue-shifted emission of the coupled nanocrystals show photon bunching at zero time delay, while emission of the uncoupled nanocrystals shows Poissonian photon statistics.

Liquid Helium Micro-PL:

Spectroscopic measurements at ~6 K were performed with a home-built microscope (DIY Cerna Components, Thorlabs). The samples were kept in a helium closed-cycle cryostat (attoDRY800, Attocube). Widefield excitation was done by 470 nm LED (EP470S10, Thorlabs) to find the regions of interest. The samples were then either excited by a pulsed laser (EPL-405, Edinburgh Instruments) at a repetition rate of 5 or 20 MHz, or a 405 nm CW laser (Wavespectrum). The lasers were focused through a cold objective (100X, 0.8 NA, Attocube), which also collected the emission. The emitted light passed through a dichroic mirror (DMLP425, Thorlabs), and a long pass filter (FELH0450, Thorlabs). The emitted light was then split with a 50:50 beamsplitter (Thorlabs) between a spectrograph (Kymera 328i, Andor) with EMCCD (iXon Ultra 888 camera, Andor), and two avalanche photodiodes (Micro Photon Devices, 100 μm PDM SPAD), with another 50:50 beamsplitter splitting the light between them. Time-stamping was performed using multichannel Time Tagger 20 (Swabian Instruments). In the lifetime measurements the spectrum was filtered to measure each peak individually (FBH500-10 and FBH520-10, Thorlabs).

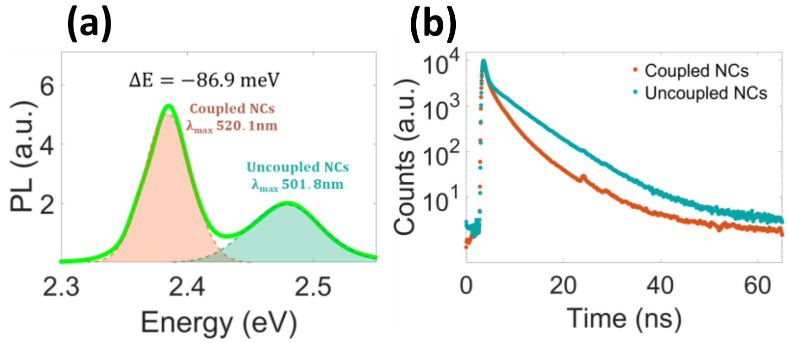

Figure S5. (a) PL spectrum at T=6K of SLs made from weakly confined 7.8nm $CsPbBr_3$ NCs. The coupled emission is red shifted. **(b)** Time resolved PL at 6K of SLs made from 7.8nm$CsPbBr_3$ NCs. Decay rates show an accelerated red-shifted coupled emission.

Excitation Power-Dependent PL:

Excitation power dependent PL measurements were conducted at 10K using 405 nm CW diode laser. The laser beam was focused using a 20x objective with NA of 0.29 and the working distance of 31 mm, reaching a laser spot diameter of 880 nm on the cooled sample. The spectrum of the emission was measured with a TRIAX 552 spectrometer equipped with a cooled CCD (Roper Scientific). Figure S6 show excitation power dependent PL of superlattices made from 7.8nm and 6.3nm $CsPbBr_3$ nanocrystals. In both samples, the peak intensity of the uncoupled emission showed a linear dependency upon the excitation power as expected, without any signs of nanocrystal degradation. The coupled red-shift PL of superlattices made from 7.8nm $CsPbBr_3$ nanocrystals showed a super-linear behavior as expected from superfluorescence, whereas the 6.3nm sample showed a coupled blue-shift emission with a sub-linear behavior. The sublinear behavior of the collective blue-shifted emission may be the result of emitter saturation in the slower collective emission mechanism at higher power or a sign of slower emission rates due to increased excitation as shown in some subradiant systems.

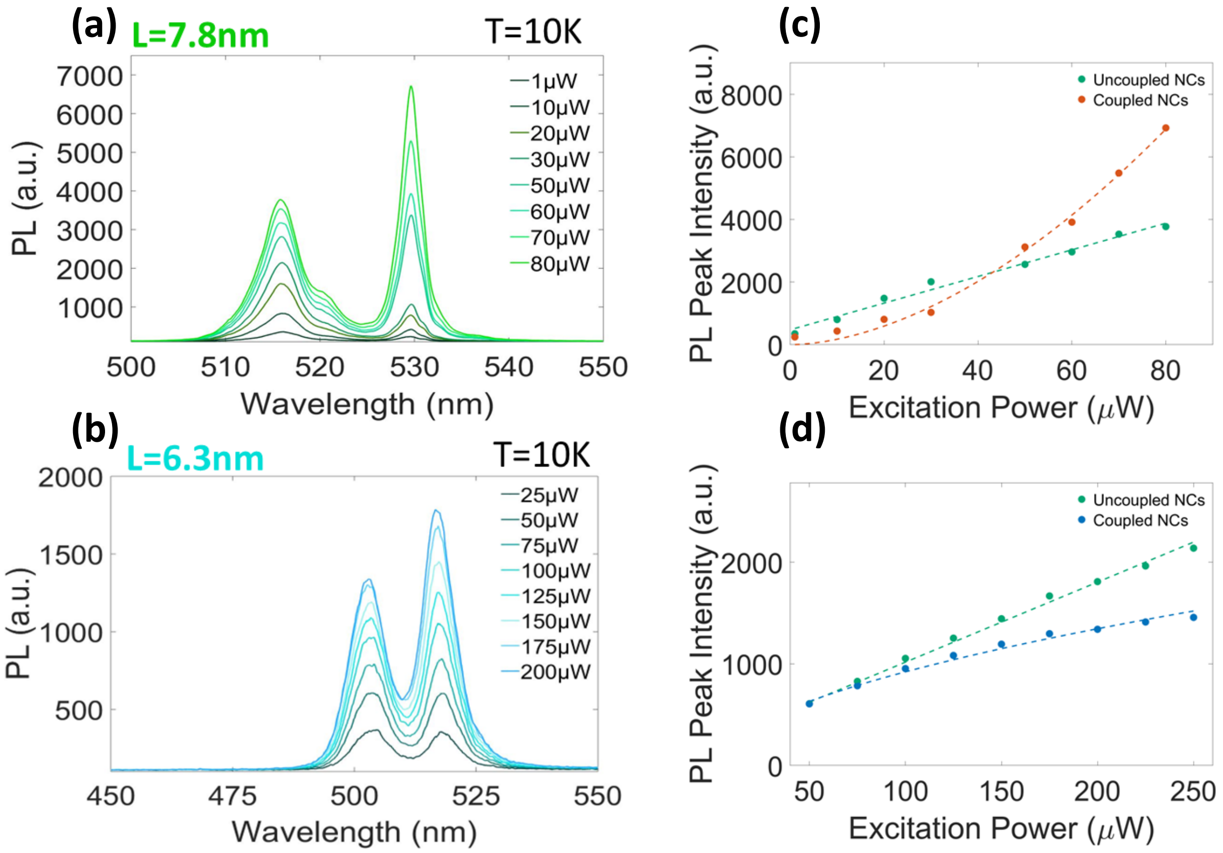

Figure S6. (a-b) Excitation power dependent PL measurements at 10K of superlattices made from (a) 7.8nm, and (b) 6.3nm $CsPbBr_3$ nanocrystals. **(c-d)** PL peak intensity of coupled and uncoupled emissions measured against excitation power of superlattices made from (c) 7.8nm, and (d) 6.3nm $CsPbBr_3$ nanocrystals. The PL of uncoupled NCs (green) behave linearly with the excitation power for both samples. The coupled red-shift PL at (c) show a super-linear behavior, and coupled blue-shift emission at (d) show a sub-linear behavior.

Energy Dispersive X-ray Spectroscopy (EDS)

SEM-EDS measurements for anion exchanged nanocrystals were carried out using an FEI Quanta200 microscope. Samples were placed at a 10 mm working distance and were measured at an acceleration voltage of 10 (KV). SEM-EDS measurements were calibrated by removing the substrate background signal of Silicon.

Table S1. EDS elemental composition results, excluding C, O, and Si.

I exchange			Cl exchange		
$CsPb(Br_{0.6}I_{0.4})_3$			$CsPb(Br_{0.6}Cl_{0.4})_3$		
Element	Line Type	Atomic %	Element	Line Type	Atomic %
Br	L series	6.6	Cl	K series	1.6
I	M series	4.5	Br	L series	2.5
Cs	M series	3.5	Cs	M series	1.3
Pb	M series	3.4	Pb	M series	1.3
$CsPb(Br_{0.7}I_{0.3})_3$			$CsPb(Br_{0.8}Cl_{0.2})_3$		
Element	Line Type	Atomic %	Element	Line Type	Atomic %
Br	L series	6.8	Cl	K series	0.8
I	M series	3.0	Br	L series	2.9
Cs	M series	3.3	Cs	M series	1.7
Pb	M series	3.1	Pb	M series	1.3

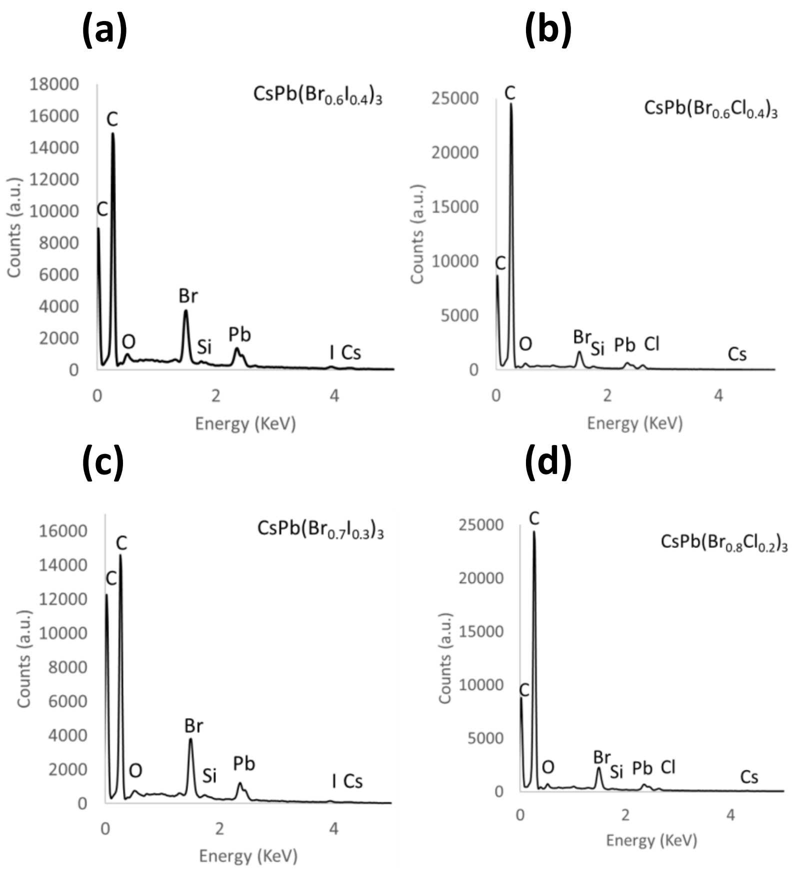

Figure S7. (a-d) EDS spectra for different samples of mixed halide from anion exchange for I exchanged (a) $CsPb(Br_{0.6}I_{0.4})_3$, and (c) $CsPb(Br_{0.7}I_{0.3})_3$. For Cl exchanges (b) $CsPb(Br_{0.6}Cl_{0.4})_3$, and (d) $CsPb(Br_{0.8}Cl_{0.2})_3$.

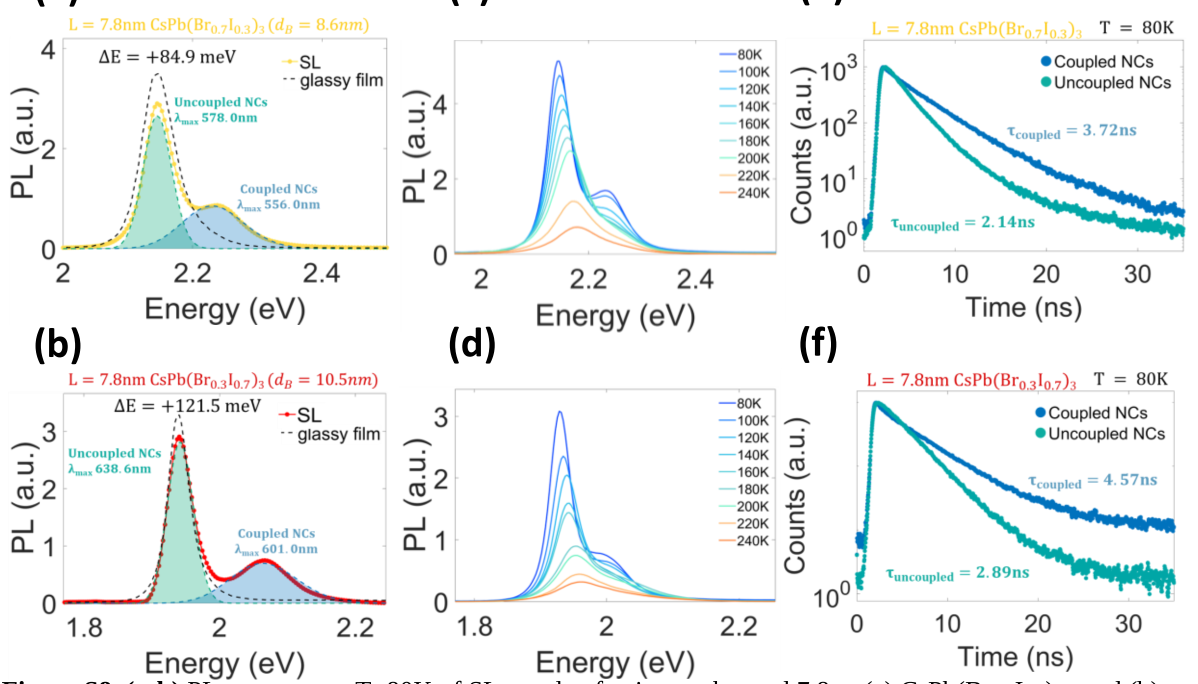

Figure S8. (a-b) PL spectrum at T=80K of SLs made of anion exchanged 7.8nm(a) $CsPb(Br_{0.7}I_{0.3})_3$, and (b) $CsPb(Br_{0.3}I_{0.7})_3$ NCs. The compositional changes modify the extent of quantum confinement and reverses the spectral behaviors of native 7.8nm $CsPbBr_3$ nanocrystal SLs. **(c-d)** PL spectra at different temperatures of SLs made of 7.8nm (c) $CsPb(Br_{0.7}I_{0.3})_3$, and (d) $CsPb(Br_{0.3}I_{0.7})_3$ NCs NCs. In the chemically strongly quantum confined NCs a blue-shifted band appear at cryogenic temperatures. **(e-f)** Time resolved PL of SL made of 7.8nm (e) $CsPb(Br_{0.7}I_{0.3})_3$, and (f) $CsPb(Br_{0.3}I_{0.7})_3$ NCs.

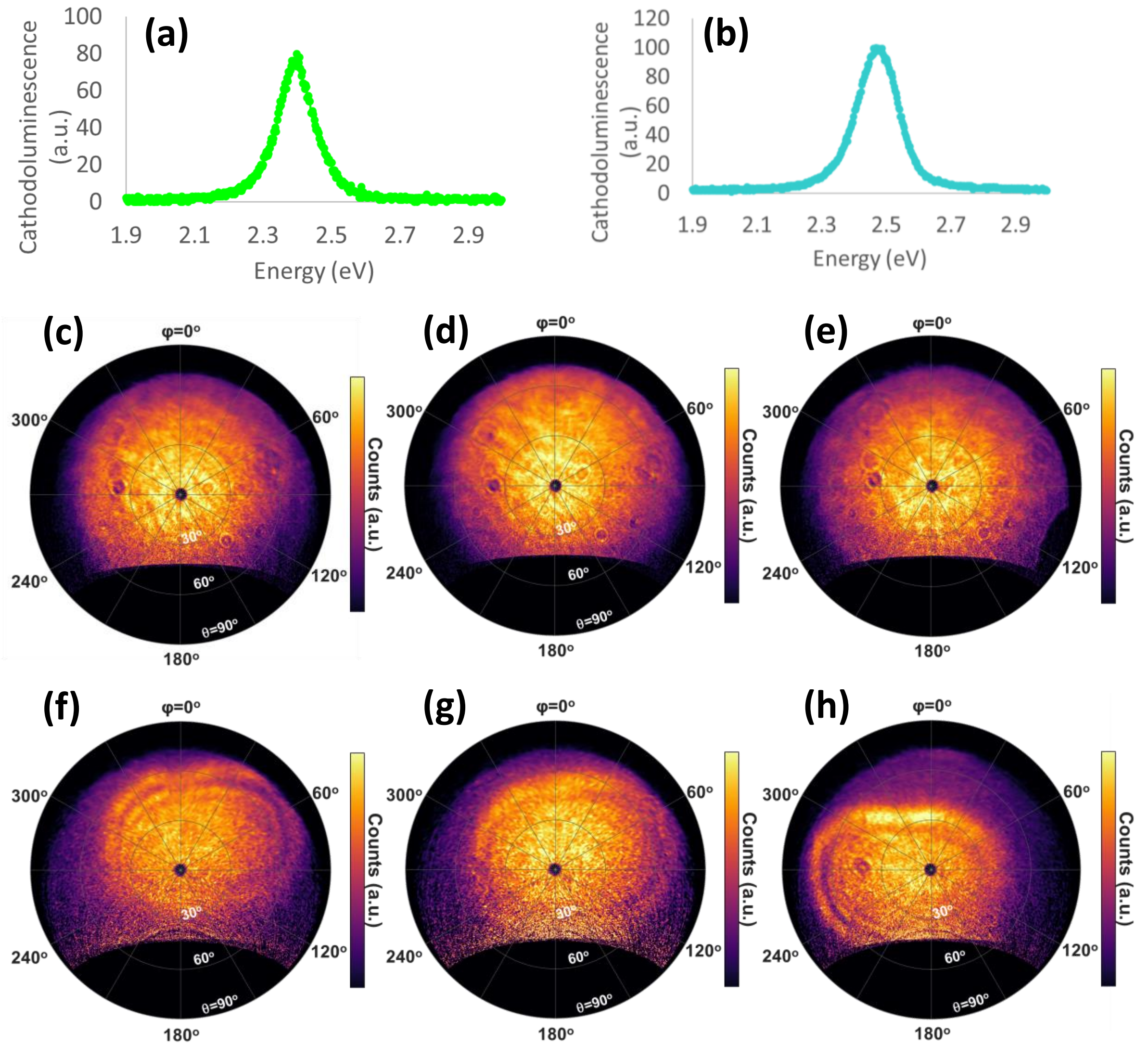

Figure S9. (a-c) CL spectra of SLs made from (a) 7.8nm, and (b) 6.3nm $CsPbBr_3$ NCs. **(c-h)** additional angular resolved CL emission patterns of SLs made from (c-e) 7.8nm, and (f-h) 6.3nm $CsPbBr_3$ NCs.

Exciton Energy Calculation

The energy of an exciton (E_{ex}) is given by:

$$[S1]\ E_{ex} = E_c + E_b$$

For E_c quantum confinement energy, and E_b binding energy. Both confinement and binding energies are dependent on the available distance in the nanocrystal, and are given by Brus equation[1]:

$$[S2]\ E_b(d) = -\frac{1.8e^2}{2\pi\varepsilon d}$$

$$[S3]\ E_c(d) = \frac{h^2}{8\left(\frac{d}{2}\right)^2}\left(\frac{1}{m_e^*} + \frac{1}{m_h^*}\right)$$

For h Planck constant, d the effective available distance for excitons in the nanocrystal, m^*_e/ m^*_h the effective mass of electrons/holes in the semiconductor, e the electron charge, and ε the effective dielectric constant of the perovskite.

For $CsPbBr_3$ the effective masses were previously reported[2]:
$m_e^* = 0.15m_0, m_h^* = 0.14m_0, \varepsilon = 4.96\varepsilon_0$ for ε_0 the vacuum permittivity.

Using these parameters, we can calculate the exciton confinement and binding energies for d=7.8nm:

$$E_c(d = 7.8\text{nm}) = \frac{(6.626 * 10^{-34})^2\left[m^4 * \frac{Kg^2}{sec^2}\right]}{8 * 3.9^2 * 10^{-18}[m^2]} * \mu\left[\frac{1}{Kg}\right]$$

$$E_c(d = 7.8\text{nm}) = 341\text{meV}$$

$$E_b(d = 7.8\text{nm}) = -\frac{1.8 * (1.6 * 10^{-19})^2[C^2]}{2\pi * 4.96 * 8.854 * 10^{-12}\left[\frac{C}{J * m}\right] * 7.8 * 10^{-9}[m]}$$

$$E_b(d = 7.8\text{nm}) = 133.6\text{meV}$$

In similar way we calculated the energy of excitons aligned at various inclination angles β in cuboid $CsPbBr_3$ nanocrystal along the edges/corners for different sized nanocrystals as presented in figure S10.

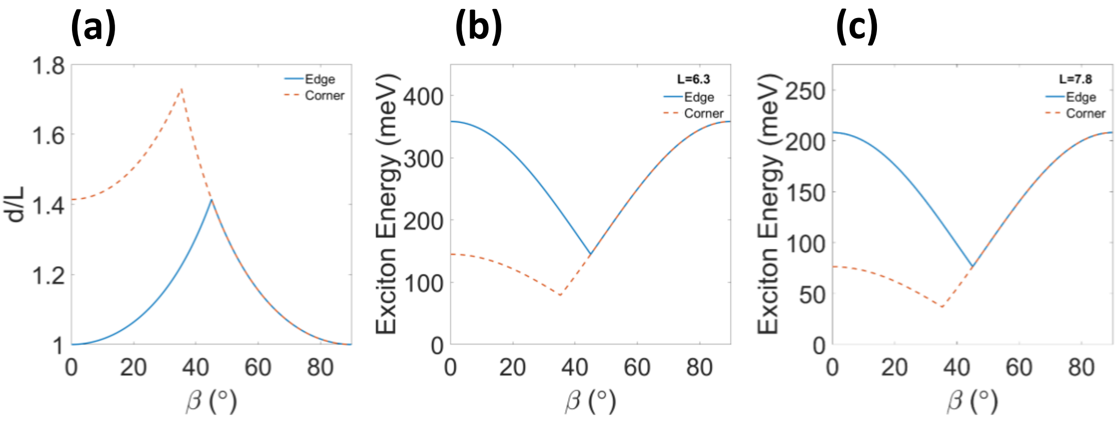

Figure S10. (a) Available effective distance in cuboid nanocrystal with edge size L for excitons aligned along the edge/corner of the nanocrystal, with inclination angle β. **(b-c)** Exciton energy for alignment along the edge/corner of the nanocrystal at various inclination angles in a cuboid nanocrystal with edge size (b) 6.3nm, and (c) 7.8nm.

X-Ray Diffraction (XRD):

One drop of dilute nanocrystal solution was cast on a silicon substrate for θ-2θ temperature-dependent measurements. Measurements were taken using a Rigaku Smart-Lab 9kW high-resolution X-ray diffractometer equipped with a rotating anode X-ray source and a HyPix-3000 detector. We used a 1.54Å (Cu Kα) wavelength and performed θ-2θ measurements with a 2θ range of 10°-70° with a step size of 0.01° and a temperature range of 298-140K. The in-situ cooling measurements were conducted using a temperature-dependent attachment stage with a carbon dome in a vacuum environment.

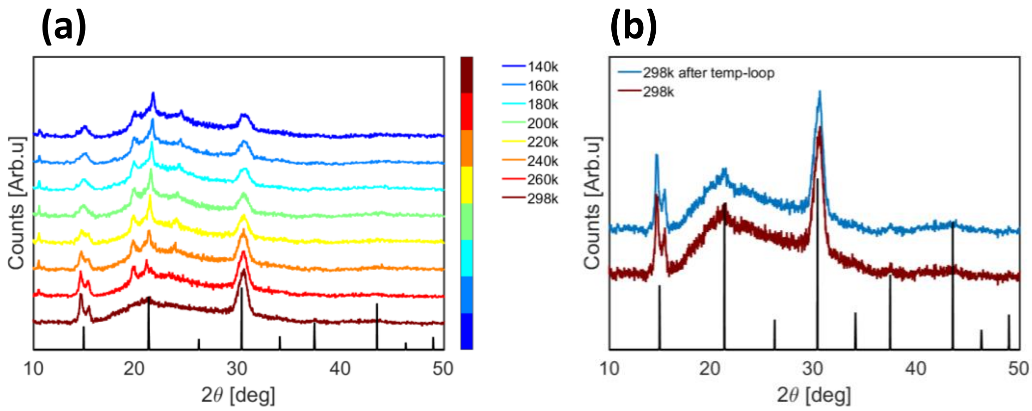

Figure S11. (a) Cryo X-ray diffractions of $CsPbBr_3$ nanocrystal SLs sample at different temperatures. **(b)** X-ray diffractions of $CsPbBr_3$ nanocrystal SL at 298K before and after cooling of the sample. Both panels include bulk reference ICDD no. 01-074-2251.

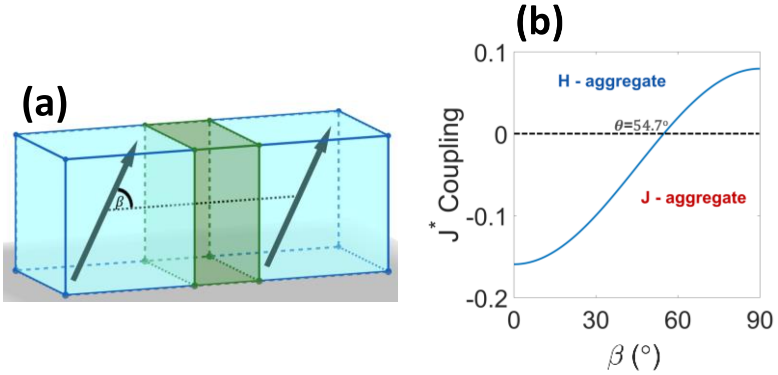

Figure S12. (a) Schematic of coupled nanocrystal dimer with coplanar transition dipole conformation. **(b)** Coulomb coupling conformation factor calculation for coplanar transition dipoles as a function of inclination angle β.

References:

1. Brus, L. E. Electron–electron and electron-hole interactions in small semiconductor crystallites: The size dependence of the lowest excited electronic state. *J Chem Phys* **80**, 4403–4409 (1984).
2. Protesescu, L. *et al.* Nanocrystals of Cesium Lead Halide Perovskites (CsPbX 3 , X = Cl, Br, and I): Novel Optoelectronic Materials Showing Bright Emission with Wide Color Gamut. *Nano Lett* **15**, 2023 (2015).
